# Amphiregulin Promotes Proliferation and Migration of the Damaged Endothelial Cells in Kawasaki Disease Cell Models

**DOI:** 10.1002/iid3.70223

**Published:** 2025-07-15

**Authors:** Jiawen Xu, Yihua Jin, Min Wang, Yijing Tao, Yujia Wang, Fangqi Gong

**Affiliations:** ^1^ Department of Cardiology, Children's Hospital, National Clinical Research Center for Child Health Zhejiang University School of Medicine Hangzhou P.R. China

**Keywords:** amphiregulin, coronary artery endothelial cells, inflammation, Kawasaki disease, macrophages

## Abstract

**Objectives:**

Amphiregulin (Areg), a member of the epidermal growth factor family, plays a critical role in tissue repair, inflammation, and immunity. Macrophages are an important source of Areg and are also among the key immune cells activated in Kawasaki disease (KD). Despite this, the role of Areg in KD has not been studied. Therefore, this study aims to investigate the expression of Areg in a KD model and to elucidate its effects on injured endothelial cells using a KD cell model.

**Methods:**

The serum of LCWE‐induced KD mouse model was measured by ELISA. RAW264.7 cells were stimulated with LCWE, and the supernatant was collected. Then, MCAECs were treated with LCWE‐induced RAW264.7 cells conditioned medium (RAW‐CM) to simulate inflammatory damage in KD endothelial cells.

**Results:**

Our study showed that the serum level of Areg increased in LCWE‐induced mouse model. In vitro, LCWE increased the expression and secretion of Areg in RAW264.7 macrophages, an effect that was inhibited by ADAM‐17 blockade. The conditioned medium (CM) from LCWE‐stimulated RAW264.7 cells (RAW‐CM) enhanced the proliferative capacity of endothelial cells, an effect that was partially inhibited by Areg antibodies. Recombinant Areg promoted the proliferation and migration of damaged endothelial cells, effects that were dependent on the activation of the AKT and ERK signaling pathways.

**Conclusion:**

This study demonstrates that serum Areg level increased in LCWE‐induced KD mouse model, and Areg promoted proliferation and migration abilities of injured endothelial cells. Our work suggests that Areg may be one of the reasons for the repair of injured endothelial cells in LCWE model vasculitis.

## 1 Introduction

Kawasaki disease (KD), first reported in 1967, is an acute self‐limited systemic vasculitis of unknown etiology with a predilection for coronary arteries. Predominantly occurring in children under 5 years of age, KD has become the leading cause of childhood‐acquired heart disease [1]. Although the etiology remains unclear, the overactivated immune system is recognized as an essential aspect of KD pathogenesis. Multiple aspects manifest the activation of the immune system in KD, such as pro‐inflammatory cells and cytokines are expanded during the acute phase of KD [2, 3, 4, 5, 6] and macrophages infiltration is found in the coronary arteries and skin lesions [7]. Vascular impairment, another important aspect of KD pathogenesis, is strongly associated with increased pro‐inflammatory cytokines and endothelial cell (EC) dysfunction [8]. In this process, activated monocytes and macrophages induce vascular ECs damage by secreting inflammatory cytokines. By chemokines, the damaged ECs recruit circulating monocytes to regions of vascular injury [9]. Recruited monocytes then differentiate into macrophages in the tissue of vascular and perivascular, further aggravating vascular damage.

Murine model of vasculitis induced by a single intraperitoneal injection of *Lactobacillus casei* cell wall extract (LCWE) mimics many of the features of human KD, including coronary artery abnormalities, aneurysms, and inflammatory cell infiltration in a coronary and pericoronary tissue [10, 11]. In the LCWE‐induced murine model of KD, the activation of macrophages and inflammatory factors is involve in the vascular impairment [12]. Hence, inflammatory responses mediated by macrophages are crucial for KD vasculitis development, whether in human or LCWE‐induced murine model.

Amphiregulin (Areg), one of the members of the epidermal growth factor family, is synthesized as membrane‐anchored precursors. After cleavage by a disintegrin and metalloproteinase 17 (ADAM‐17), Areg work as an autocrine or paracrine factor [13]. Areg has great impact in tissue repair, suppression of inflammation, and immunity. Literatures indicate activated macrophages are a critical source of Areg [14, 15]. Considering macrophages have important functions in KD vasculitis, our objective is to investigate the impact of macrophage‐derived Amphiregulin on KD vasculitis. Hence, this study aims to conduct an initial exploration on expression and role of Areg in KD cell models.

In this study, we initially assessed the serum concentrations of Areg in a KD murine model, with the aim of exploring the potential association between Areg expression and vasculitis. Afterward, we mimicked KD artery ECs injury in vitro by treating mouse coronary artery endothelial cells (MCAECs) with LCWE‐treated RAW264.7 cells conditioned medium (RAW‐CM), as reported in previous studies [12]. ADAM‐17 inhibitors, recombinant mouse Areg, and Areg blocking antibody were used in KD cell model, to investigate the possible role and mechanisms of Areg in orchestrating ECs damage in KD. Our results showed that serum levels of Areg increased in the early stages of KD murine model, and Areg promotes proliferation and migration of damaged EC by AKT and ERK signaling pathways.

## 2 Materials and Methods

### 2.1 LCWE‐Induced KD Mouse Model

#### 2.1.1 Mice

Five‐week‐old male C57BL/6 mice were purchased from Slake Laboratory Animal Co. Ltd. (Shanghai, China) and housed under specific pathogen‐free conditions at the animal center of Zhejiang Chinese Medical University. All animal experiments were approved by the Animal Ethical and Welfare Committee of Zhejiang Chinese Medical University (Approval Number: IACUC‐20230717‐04) and conducted in accordance with the guidelines of the Animal Center.

#### 2.1.2 LCWE and Experimental Grouping

The LCWE sample was synthesized and its concentration determined by referring to previous methods [16]. Then, 16 mice were randomly divided into two groups (*n* = 8): LCWE group and control group. LCWE group mice were injected intraperitoneally with 500 μL LCWE (2 mg/mL in PBS). Control group was injected with 500 μL PBS. Mice were euthanized for tissue harvest on day 1 or 14 postinjection. Blood samples were obtained through the inferior vena cava puncture, from 4 mice per group on Day 1. After clotting at room temperature for 2 h, all blood samples were centrifuged for 20 min at 1000 g to separate the serum. Then, the serum samples were stored at −80°C immediately until analysis. Subsequently, the expression levels of Areg, IL‐6, IL‐1β, TNF‐α, and MCP‐1 in serum were detected by ELISA. The heart tissues (at day 14 postinjection) were perfused with PBS and then immersed in 4% paraformaldehyde for over 24 h. Subsequent procedures including embedding, sectioning H&E staining were submitted to Pinuofei Biological Technology Co. Ltd (Wuhan, China).

#### 2.1.3 Immunofluorescence Analysis

Paraffin‐embedded heart tissue sections were deparaffinized and subjected to heat‐mediated antigen retrieval, followed by H_2_O_2_ for 30 min. Then, the sections were blocked in PBS with 3% BSA at room temperature for 30 min, and incubated with the primary antibodies against F4/80 (CST, Cat. No. 70076S) or Areg (huabio, Cat. No. ER1903‐67) at 4°C overnight. Next, the sections were washed and incubated with goat anti‐rabbit FITC (Proteintech, Cat. No. SA00003‐2) or goat anti‐rabbit Cy3 (Proteintech, Cat. No. SA00009‐2) at room temperature in the dark for 1 h. Mounting Medium containing DAPI (Vector Labs, Burlingame, CA, USA) was used to mount sections. Images were captured with Leica fluorescence microscope.

### 2.2 EC Injury Model In Vitro

#### 2.2.1 Cell Culture and Grouping

Both RAW264.7 cells and primary MCAECs were purchased from Procell Life Science and Technology Co. Ltd (Wuhan, China). RAW264.7 cells were cultured in complete DMEM (Procell), containing 10% fetal bovine serum (FBS) and 1% penicillin‐streptomycin (P/S) solution. MCAECs were cultured in MCAEC‐specialized medium (Procell) containing 1% endothelial cell growth supplement, 5% FBS, and 1% P/S solution. Cells were incubated at 37°C with 5% CO_2_ atmosphere in a humidified incubator, and the culture medium was changed every 24 h (RAW264.7 cells) or 48 h (primary MCAECs). When 70%–80% confluent, RAW264.7 or primary MCAECs were passaged or seeded into 96‐, 24‐, or 6‐well plates for subsequent experiments.

RAW264.7 cells in well plates were grouped: Control group, LCWE group, TAPI‐1 group, and LCWE + TAPI‐1 group. The treatments were as follows: After RAW264.7 cells became adherent, the medium was exchanged with basic DMEM medium (Gibco, Grand Island, NY, USA). And then RAW264.7 cells were stimulated by LCWE (1 μg/mL) for 12 h with or without pretreatment with TAPI‐1 (ADAM17 inhibitor, MCE, Shanghai, China) at different concentrations (0, 1, 5, 10, and 15 μΜ) for 30 min.

The RAW‐CM was collected and centrifugated at 1000 × g for 5 min to remove cell debris. MCAECs were incubated with RAW264.7‐CM to establish the MCAECs injury model as previously described [12]. The MCAECs were grouped: Control group, LCWE group, LCWE + TAPI‐1 group (5 μM), Areg group, Areg + LCWE group, Anti‐Areg group, Anti‐Areg + LCWE group and IgG + LCWE group. The treatments were as follows: Control group was cultured with basic DMEM medium. LCWE group and LCWE + TAPI‐1 were incubated with RAW264.7‐CM and RAW264.7 + TAPI‐1‐CM. Areg group and Areg + LCWE group were treated with recombinant mouse Areg (rm‐Areg; 1 μg/mL; R&D System, MN, USA) in basic DMEM medium and RAW264.7‐CM. RAW‐CM was pretreated with Areg blocking antibody (anti‐Areg; 5 μg/mL; R&D System, MN, USA) or IgG (5 μg/mL; R&D System, MN, USA) for 2 h to block intrinsic secreted Areg, then cultured MCAECs as Anti‐Areg + LCWE group and IgG + LCWE group.

#### 2.2.2 Cell Viability Assay

Cell viability was measured using the cell counting kit 8 (CCK‐8) assay. RAW264.7 cells (1 × 10^4^ cells/well) or MCAECs (5 × 10^3^ cells/well) were seeded into 96‐well plates overnight. The following day, the old medium was changed to basic DMEM medium. RAW264.7 cells treated with or without TAPI‐1 (0, 1, 5, 10, and 15 μΜ) for 30 min, then stimulated or left unstimulated by LCWE (1 μg/mL) for 12 h. MCAECs were incubated with RAW‐CM from different treatments (LCWE, LCWE + TAPI‐1, LCWE + Areg, LCWE + anti‐Areg, LCWE + IgG) as mentioned above for 12 h. Then 10 μL CCK‐8 (APExBIO, Houston, TX, USA) solution was added to each culture well and incubated at 37°C for 4 h. Absorbance at 450 nm was measured using a microplate reader (Bio‐Tek, Winooski, VT, USA).

#### 2.2.3 Reverse Transcription—Quantitative Real‐Time PCR

According to the manufacturer's instructions, the total RNA was extracted from RAW264.7 cells or MCAECs using the EZ‐press RNA Purification Kit (EZBioscience, Roseville, CA, USA, Cat. No. B0004D), and cDNA was synthesized using the MonScript RTIII All‐in‐One Mix with dsDNase kit (Monad, Wuhan, China, Cat. No. MR05101S).

Real‐time quantitative PCR (qPCR) was performed using the ABI‐7500 StepOne Plus Real‐Time PCR System (Applied Biosystems, Foster City, CA, USA) with the ChamQ SYBR qPCR Master Mix kit (Vazyme, Nanjing, China, Cat. No. Q431‐02). The qPCR reaction was conducted in a total volume of 20 µL, containing 10 µL of 2× ChamQ SYBR qPCR Master Mix, 1 µL of forward primer (0.2 µM final concentration), 1 µL of reverse primer (0.2 µM final concentration), 2 µL of cDNA template, and 6 µL of RNase‐free water. The sequences of the forward and reverse primers are provided in Table 1.

**Table 1:** The primers used for RT‐qPCR in this study.

Gene	Forward 5ʹ‐3ʹ	Reverse 5ʹ‐3ʹ
GAPDH	AGGTCGGTGTGAACGGATTTG	TGTAGACCATGTAGTTGAGGTCA
Areg	GCAGATACATCGAGAACCTGG	CTGCAATCTTGGATAGGTCCTTG
TNF‐α	CAGGCGGTGCCTATGTCTC	CGATCACCCCGAAGTTCAGTAG
IL‐6	GAGGATACCACTCCCAACAGACC	AAGTGCATCATCGTTGTTCATACA
IL‐1β	GCAACTGTTCCTGAACTCAACT	ATCTTTTGGGGTCCGTCAACT
MCP‐1	TAAAAACCTGGATCGGAACCAAA	GCATTAGCTTCAGATTTACGGGT

Abbreviations: Areg, amphiregulin; GAPDH, glyceraldehyde‐3‐phosphate dehydrogenase; IL‐1β, interleukin‐1β; IL‐6, interleukin‐6; MCP‐1, monocyte chemotactic protein‐1; TNF‐α, tumor necrosis factor‐α.

The thermal cycling conditions were as follows: initial denaturation at 95°C for 30 s, followed by 40 cycles of 95°C for 10 s and 60°C for 30 s. Melting curve analysis was performed immediately after amplification to confirm the specificity of the PCR products. The dissociation curve program included an initial hold at 95°C for 15 s, followed by a hold at 60°C for 1 min, and a temperature ramp from 60°C to 95°C, increasing by 0.05°C per step with a hold of 5 s per step. Fluorescence data were collected during the temperature ramp to generate the melting curve.

The relative mRNA levels were normalized to the reference gene GAPDH and calculated using the 2^(−ΔΔCt) method.

#### 2.2.4 Wound Healing Assay (Scratch Assay)

Wound‐healing assay was used to evaluate the migration capacity of injured MCAECs in different conditions. After MCAECs reached 90% confluency in six‐well plates, the medium was changed to LCWE + TAPI‐1‐treated RAW‐CM or LCWE‐treated RAW‐CM, with or without 5 μg/mL anti‐Areg. After 12 h, linear scratch was done using a sterile 200 μL tip and washed with PBS. Injured MCAECs were further cultured in basic DMEM medium with or without 1 μg/mL Areg. Culture medium was replaced at 36 h. The scratch gap was recorded and photographed with a light microscope at 0 and 72 h. ImageJ software (NIH, USA) was used to measure scratch areas. Cell migration capacity was reported as % of wound area coverage by calculating the ratio of the scratch gap area at 72 and 0 h.

#### 2.2.5 Western Bloting

Total proteins were extracted by RIPA Lysis Buffer (Beyotime Biotechnology, Shanghai, China) containing HaltTM Protease and Phosphatase Inhibitor Cocktail (Thermo Scientific, Rockford, IL, USA). Protein concentration was determined by Pierce BCA Protein Assay Kit (Thermo Scientific, Rockford, IL, USA). Equal amounts of total protein were separated on 10% SDS‐polyacrylamide gel electrophoresis, then transferred onto PVDF membranes (Merck Millipore, Darmstadt, Germany). The membranes were blocked in TBST with 5% nonfat milk for 1 h at room temperature, and incubated with the primary antibodies at 4°C overnight. Next, the membranes were incubated with corresponding horseradish peroxidase‐conjugated secondary antibodies for 1 h at room temperature. Target protein bands were visualized by ECL reagents (FDbio Science, Hangzhou, China). Band density was quantified using ImageJ software. Primary antibodies against AKT (Cat. No. 4691), Phospho‐AKT‐Thr308 (Cat. No. 13038), Phospho‐AKT‐Ser473 (Cat. No. 4060), ERK1/2 (Cat. No. 4695), and Phospho‐ERK1/2‐Thr202/Tyr204 (Cat. No. 4370) were purchased from Cell Signaling (Danvers, MA, USA). Primary antibodies against GAPDH (Cat. No. 60004‐1‐Ig) and ADAM‐17 (Cat. No. 29948‐1‐AP) were purchased from Proteintech (Wuhan, China). Goat anti‐rabbit (Cat. No. ab205718) and goat anti‐mouse (Cat. No. ab205719) secondary antibodies were purchased from Abcam (Cambridge, MA, USA).

#### 2.2.6 Establishment of a Stable Cell Line

Short hairpin RNAs (shRNAs) were used to silence ADAM‐17 (sh‐ADAM17) and were transfected into 293 T cells together with the psPAX2 and pMD2G auxiliary plasmids to package the lentivirus. RAW264.7 cells were infected with the lentivirus (MOI:50) for 48 h, then 6 μg/mL puromycin was used for 72 h. The stable cell line was then used for the subsequent experiments. The lentivirus was obtained from Hanbio Company (Shanghai, China), and the shRNA sequences are listed in Table 2.

**Table 2:** Short hairpin RNA sequences.

Short hairpin RNA	Top strand sequence	Bottom strand sequence
NC	GATCCGTTCTCCGAACGTGTCACGTAATTCAAGAGATTACGTGACACGTTCGGAGAATTTTTTC	AATTGAAAAAATTCTCCGAACGTGTCACGTAATCTCTTGAATTACGTGACACGTTCGGAGAACG
sh‐ADAM17#1	GATCCGAGTGCAGATAGAGCAGATTCGAATTTCAAGAGAATTCGAATCTGCTCTATCTGCACTCTTTTTTG	AATTCAAAAAAGAGTGCAGATAGAGCAGATTCGAATTCTCTTGAAATTCGAATCTGCTCTATCTGCACTCG
sh‐ADAM17#2	GATCCGCAAACTGCAGTAAACAGTCCATCTATTCAAGAGATAGATGGACTGTTTACTGCAGTTTGTTTTTTG	AATTCAAAAAACAAACTGCAGTAAACAGTCCATCTATCTCTTGAATAGATGGACTGTTTACTGCAGTTTGCG
sh‐ADAM17#3	GATCCGGAGGCTATTAATGCTACATGCAAATTCAAGAGATTTGCATGTAGCATTAATAGCCTCCTTTTTTG	AATTCAAAAAAGGAGGCTATTAATGCTACATGCAAATCTCTTGAATTTGCATGTAGCATTAATAGCCTCCG

### 2.3 Enzyme‐Linked Immunosorbent Assay (ELISA)

The cytokine concentrations include human Areg, murine Areg, murine IL‐1β (ELK Biotech, China), murine MCP‐1, murine IL‐6, and murine TNF‐α (Absin, China) in serum or cell culture medium were measured by commercial ELISA Kit according to the manufacturer's instructions.

### 2.4 Statistics

All the data were analyzed using SPSS (version 26.0) software or GraphPad Prism (version 8.0) software, and expressed as mean ± SD unless special illustration. The two‐tailed unpaired *t*‐test was used to compare the two groups. Multiple groups were compared using single‐factor ANOVA. Statistically significance was defined as *p* values of < 0.05 (* or #), < 0.01 (** or ##), < 0.001 (*** or ###) or < 0.0001 (**** or ####). Each experiment was repeated at least three times.

## 3 Results

### 3.1 Serum Amphiregulin Levels Increased in LCWE‐Induced KD Vasculitis Model

**Figure 1:**
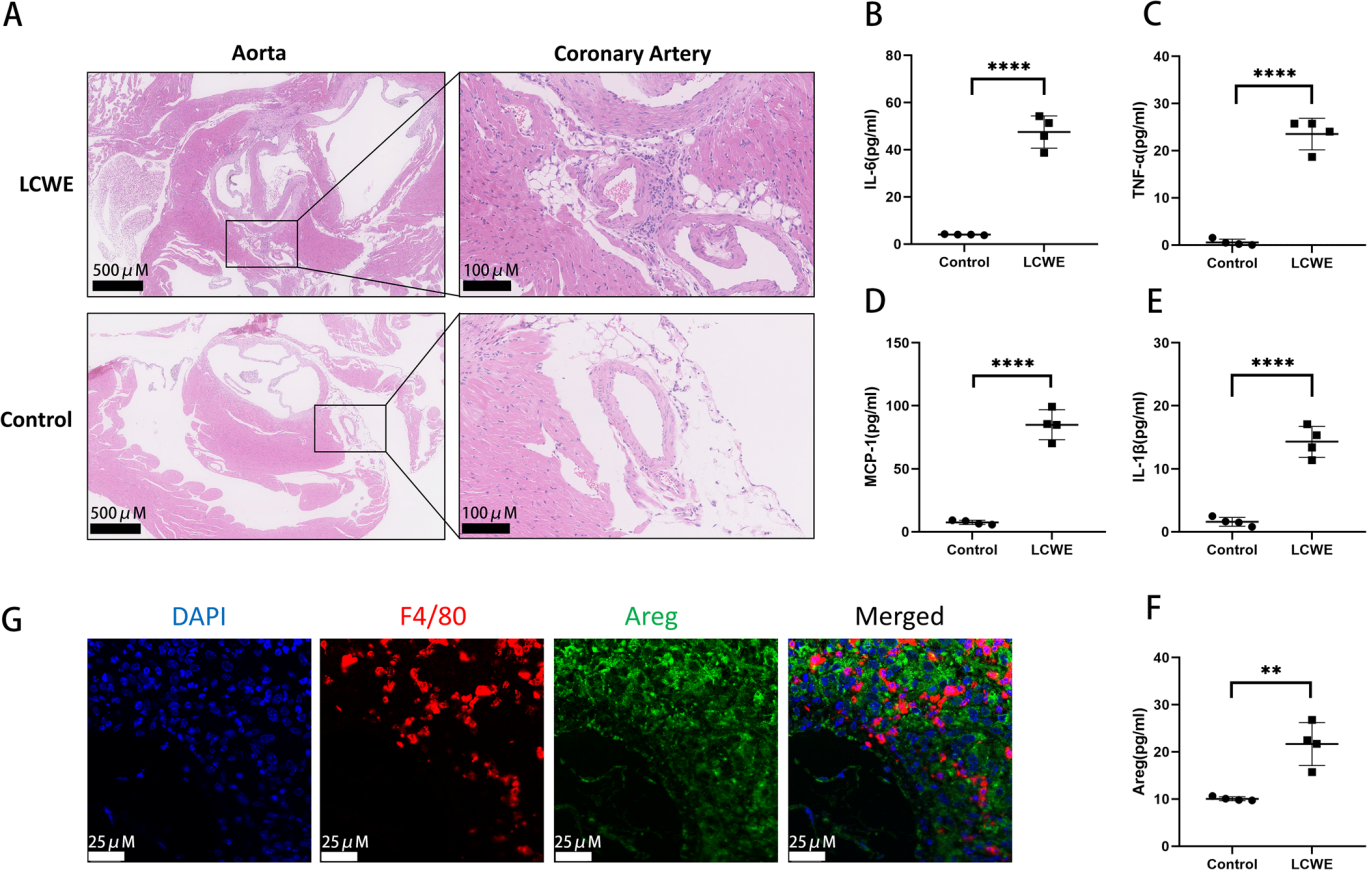
Serum levels of cytokines increased in LCWE‐induced murine model. (A) HE‐stained heart sections of LCWE and control group mice. Serum levels of IL‐6 (B), TNF‐α (C), MCP‐1 (D), IL‐1β (E), and Areg (F) in LCWE‐induced murine model were measured by ELISA. (G) Pericoronary tissue of KD murine model stained for F4/80 (red) and Areg (green) by IF analysis. Data are presented as mean ± SD, with each data point representing an independent measurement. ****p < 0.0001, **p < 0.01 versus the control group.

To elucidate the expression profile of Amphiregulin (Areg) within the context of the KD model, we established an LCWE‐induced vasculitis model. Coronary vasculitis and systemic inflammation in the mouse models were detected in this part of study. In LCWE group, the tissue surrounding the aortic root and the coronary arteries showed a profound inflammatory response in 14 days after LCWE injection (Figure 1A). The inflammatory factors, including IL‐6, TNF‐α, MCP‐1, and IL‐1β (Figure 1B–E) were significantly increased 24 h after LCWE injection. Amphiregulin (Figure 1F) increased in LCWE group compared to control group. Double IF staining was performed using paraffin sections of KD murine model (Figure 1G). F4/80 is a marker for macrophages. The results revealed that macrophages and Areg were widely expressed in pericoronary tissue.

### 3.2 TAPI‐1 Suppressed LCWE‐Induced Amphiregulin and Inflammatory Cytokines Expression in RAW264.7 Cells

**Figure 2:**
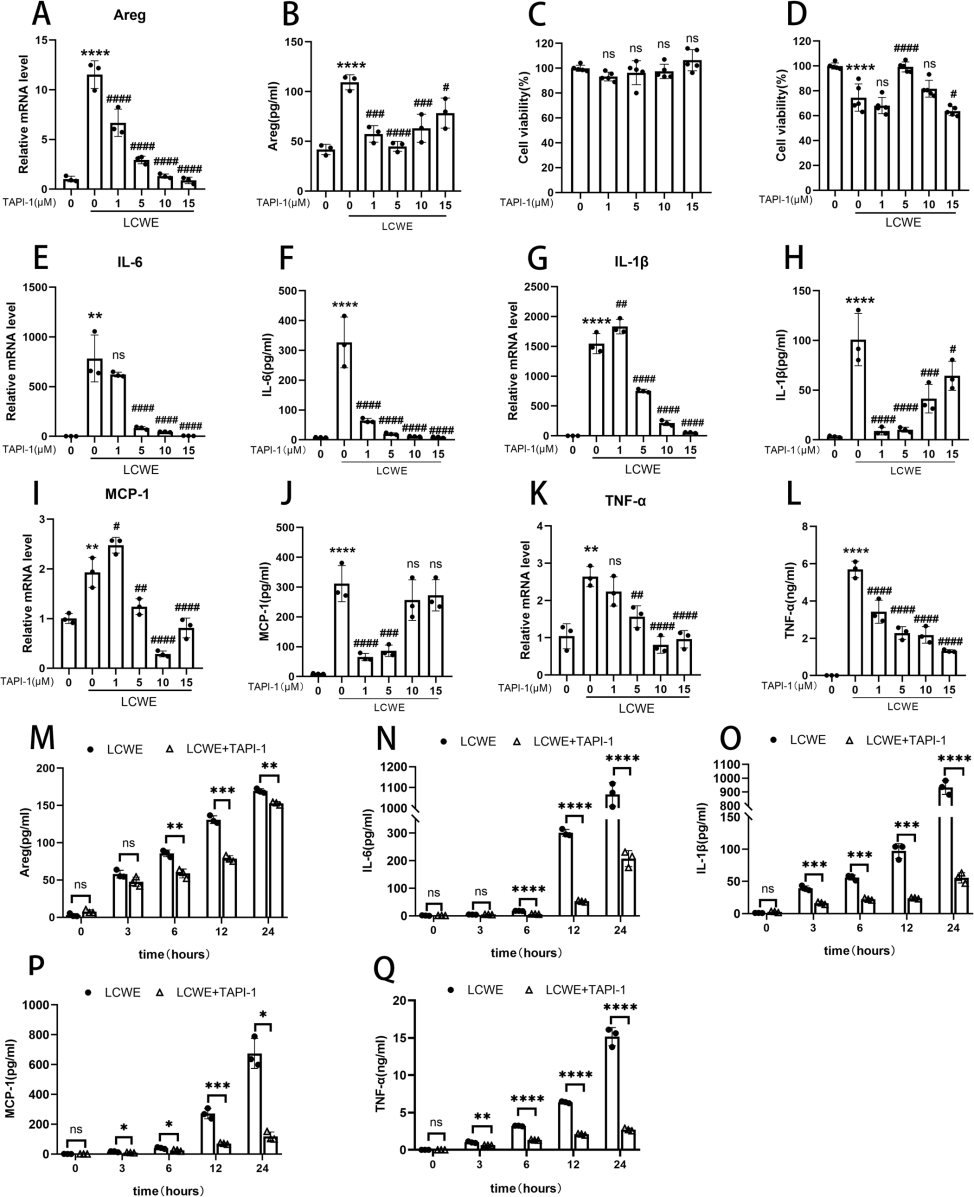
TAPI‐1 inhibits expression of Areg, IL‐6, IL‐1β, MCP‐1, and TNF‐α in LCWE‐treated RAW264.7 cells. RAW264.7 cells were pretreated with TAPI‐1 (0, 1, 5, 10, 15 μM) 30 min, then stimulated or unstimulated with LCWE (1 μg/mL) for 12 h. (A, B) Cell viability of RAW264.7 cells was measured by CCK8 assay. The mRNA and protein expression levels of Areg (C, D), IL‐6 (E, F), IL‐1β (G, H), MCP‐1 (I, J), and TNF‐α (K, L) were assayed by qRT‐PCR and ELISA, respectively. Results are expressed as the mean ± SD, **p < 0.01, ****p < 0.0001 versus untreated group; #*p* < 0.05, ##*p* < 0.01, ###*p* < 0.001, ####*p* < 0.0001 versus 0 μM TAPI‐1 LCWE group. Five micromolar TAPI‐1 inhibits levels of secreted Areg (M), IL‐6 (N), IL‐1β (O), MCP‐1 (P), and TNF‐α (Q) at indicated time points (0, 3, 6, 12, 24 h). Results are expressed as the mean ± SD, *p < 0.05, **p < 0.01, ***p < 0.001, ****p < 0.0001.

Subsequently, we treated RAW264.7 macrophages with LCWE to further explore the elevation mechanism and role of Areg in vitro. ELISA and qRT‐PCR were used to detect amphiregulin expression in RAW264.7 macrophages in different treatments. The results showed that amphiregulin protein and mRNA levels were increased conspicuously at 12h after LCWE stimulation, which inhibited by pretreating with TAPI‐1 (Figure 2A,B). TAPI‐1 is an ADAM‐17 inhibitor that blocks amphiregulin expression and secretion. The effect of TAPI‐1 on RAW264.7 cell viability was evaluated by CCK8 assay. The results showed that TAPI‐1 has no significant toxicity at a concentration of ≤ 10 µM (Figure 2C,D). Gene expressions of IL‐6, IL‐1β, MCP‐1, and TNF‐α were detected by qRT‐PCR. The results showed LCWE promoted the transcript levels of IL‐6, IL‐1β, MCP‐1, and TNF‐α in RAW264.7 cells, which were suppressed by TAPI‐1 at a concentration between 5 and 15 µM (Figure 2E,G,I,K), at 12 h after LCWE stimulation. ELISA evaluated protein secretion levels of IL‐6, IL‐1β, MCP‐1, and TNF‐α. The results showed LCWE promoted the secretion of IL‐6, IL‐1β, MCP‐1, and TNF‐α in RAW264.7 cells, which was suppressed by TAPI‐1 at a concentration between 1 and 5 µM (Figure 2F,H,J,L).

### 3.3 Knocking Down ADAM‐17 Inhibits Expression of Areg, IL‐6, and TNF‐α

**Figure 3:**
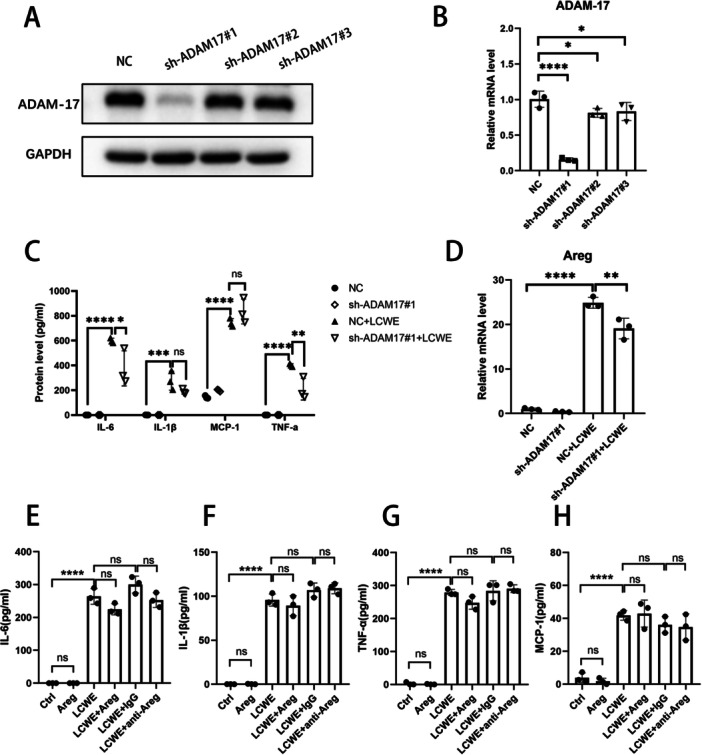
Knocking down ADAM‐17 inhibits expression of IL‐6 and TNF‐α. (A and B) Three lentivirus shRNAs (sh‐ADAM17#1, sh‐ADAM17#2, or sh‐ADAM17#3) were introduced into RAW264.7 cells, and the mRNA and protein expression levels of ADAM‐17 measured by WB and qRT‐PCR. (C) NC and sh‐ADAM17#1 RAW264.7 cells were treated with LCWE (1 μg/mL) for 12 h, the protein expression levels of IL‐6, IL‐1β, MCP‐1, and TNF‐α were assayed by ELISA. (D) The mRNA expression level of Areg was measured by qRT‐PCR. (E–H) LCWE‐stimulated RAW264.7 cells were treated with or without exogenous rm‐Areg (1 μg/mL), anti‐Areg (5 μg/mL), or IgG (5 μg/mL) for 12 h, then the protein expression levels of IL‐6, IL‐1β, MCP‐1, and TNF‐α were assayed by ELISA. Results are expressed as the mean ± SD, *p < 0.05, **p < 0.01, ***p < 0.001, ****p < 0.0001.

To further determine the inhibition of ADAM‐17 on inflammatory cytokines and Areg. ADAM‐17 was knocked down by lentivirus shRNAs. Three lentivirus ADAM‐17 shRNAs (sh‐ADAM17#1, sh‐ADAM17#2, or sh‐ADAM17#3) were introduced into RAW264.7 cells. As shown in Figure 3A,B, sh‐ADAM17#1 RAW264.7 cells exhibited the strongest level of ADAM‐17 inhibition. Therefore, sh‐ADAM17#1 RAW264.7 cells were used in the subsequent studies. The ELISA result revealed that ADAM‐17 inhibition significantly suppressed the expressions of IL‐6 and TNF‐α, but there was no significant effect on IL‐1β and MCP‐1 expressions (Figure 3C). And ADAM‐17 inhibition significantly suppressed the expressions of Areg (Figure 3D). To further determine the effects of ADAM‐17 downstream molecule Areg on inflammatory factors. Exogenous rm‐Areg, anti‐Areg, or IgG were used in LCWE‐stimulated RAW264.7 cells for 12 h, respectively. ELISA was used to detect the expression of inflammatory factors. As shown in Figure 3E–H, Areg had no significant effect on expressions of IL‐6, IL‐1β, MCP‐1, and TNF‐α. Therefore, in the ADAM17‐Areg pathway, only the upstream ADAM17 can upregulate the expression of inflammatory cytokines IL‐6 and TNF‐α.

### 3.4 Exogenous Areg Facilitated the Proliferation and Migration of Injured MCAECs via AKT and ERK Pathways

**Figure 4:**
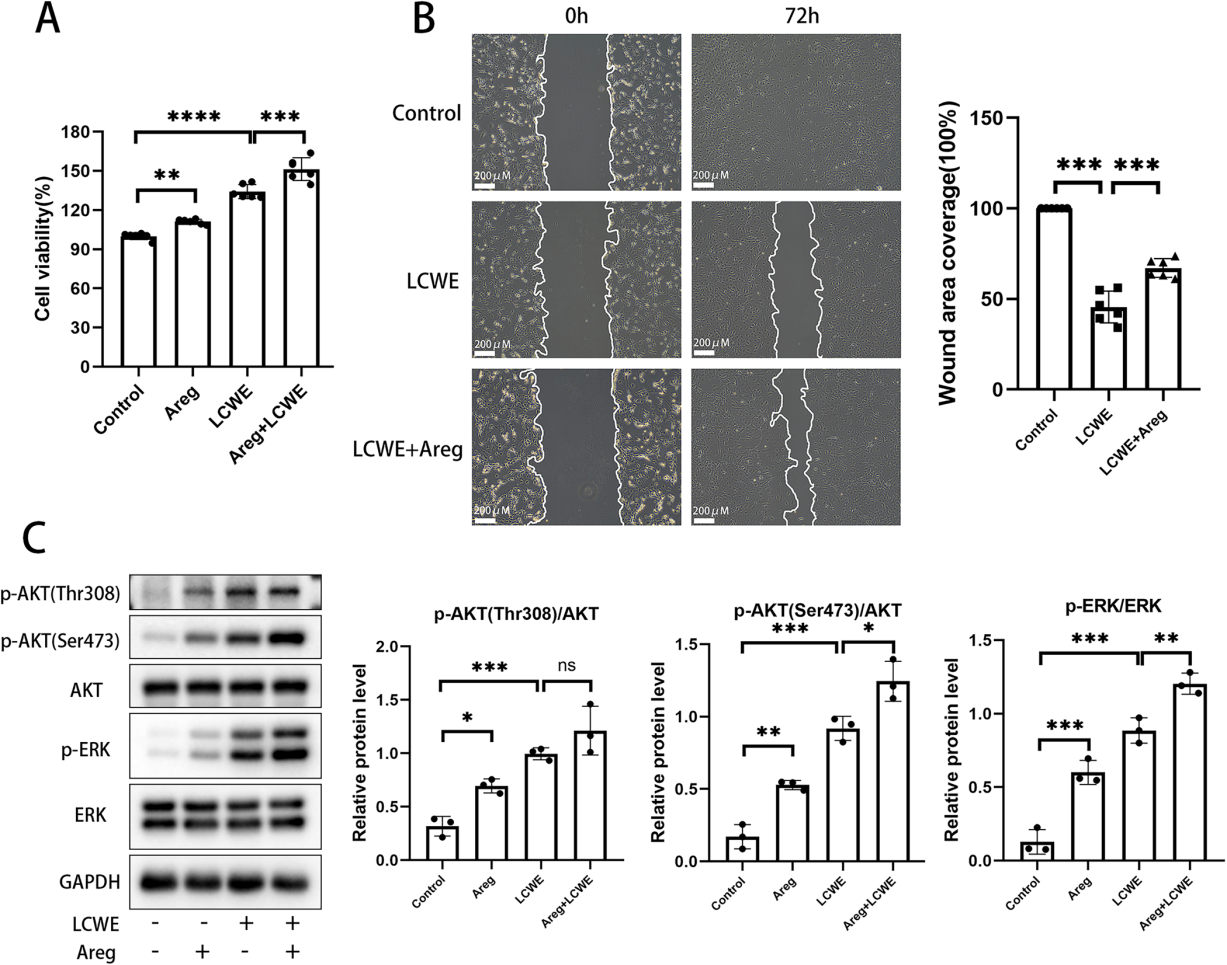
Exogenous Areg promotes the proliferation and migration of injured MCAECs. MCAECs were incubated with LCWE‐treated RAW‐CM, with or without exogenous rm‐Areg (1 μg/mL). After 12 h stimulation, cell viability (A) was measured using CCK8 assay, and cell migration (B) was determined by Wound‐Healing assay. (C) After treatment with RAW‐CM for 1 h, phosphorylation level of AKT and ERK in MCAECs were examined by Western blot. Data are presented as mean ± SD, *p < 0.05, **p < 0.01, ***p < 0.001, ****p < 0.0001.

Endothelial lesion is an important part of vascular injury in KD. MCAECs were treated with LCWE‐induced RAW‐CM to simulate inflammatory damage in KD ECs. Proliferation and migration are important functions of ECs. The result of CCK8 assay showed exogenous recombinant mouse Areg dramatically increased the cell viability of MCAECs with or without LCWE‐treated RAW‐CM culture (Figure 4A). Based on the wound‑healing assay, exogenous Areg significantly augmented the migratory ability of injured MCAECs (Figure 4B). The result of western blotting showed that exogenous Areg notably promoted the levels of AKT and ERK phosphorylation in LCWE‐mediated injured MCAECs (Figure 4C).

### 3.5 Blocking Endogenous Areg Lessened the Proliferation and Migration of Injured MCAECs via AKT and ERK Pathways

**Figure 5:**
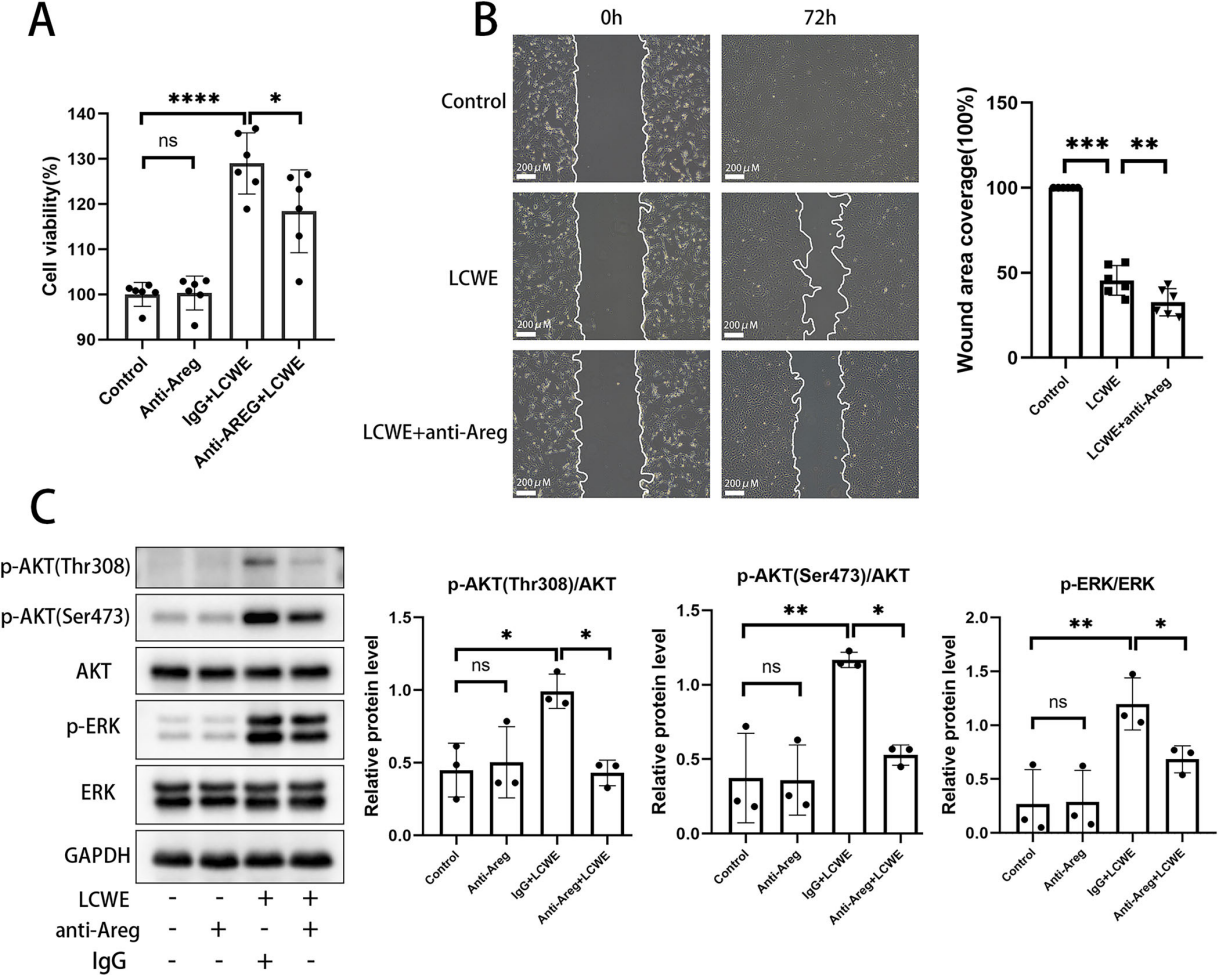
Blocking intrinsic secreted Areg by antibody inhibits the proliferation and migration of injured MCAECs. LCWE‐treated RAW‐CM was pretreated with anti‐Areg (5 μg/mL) or IgG (5 μg/mL) for 2 h. Then, MCAECs were incubated with the pretreated RAW‐CM or only exposed to anti‐Areg (5 μg/mL). After culture for 12 h, cell viability (A) was measured by CCK8 assay, and cell migration (B) was determined by Wound‐Healing assay. (C) Phosphorylation level of AKT and ERK after 1 h‐incubation were examined by Western blot. Data are presented as mean ± SD, *p < 0.05, **p < 0.01, ***p < 0.001, ****p < 0.0001.

The outcome of the CCK8 assay demonstrated that anti‐Areg significantly attenuated the proliferation of MCAECs promoted by LCWE‐treated RAW‐CM (Figure 5A). In addition, the findings of the wound healing assay indicated that anti‐Areg further suppressed the migratory capacity of injured MCAECs (Figure 5B). The result of western blotting showed that endogenous Areg blockage significantly reduced the levels of AKT and ERK phosphorylation in LCWE‐mediated injured MCAECs (Figure 5C).

## 4 Discussion

KD is a systemic vasculitis predominantly affecting children under 5 years of age and has become the leading cause of acquired heart disease in children. The overactivated immune response is the most important clinical feature of acute KD [17], characterized by the infiltration of inflammatory cells and elevated levels of pro‐inflammatory cytokines. Our study aims to explore the role of Areg in KD, particularly in the context of EC injury and repair.

First, to elucidate the expression and role of Areg in KD, we established both an animal model and a cell model of KD. KD is widely recognized as an immune‐mediated inflammatory cascade, typically triggered by uncertain stimuli or infectious agents in children with genetic susceptibility. This condition is characterized by the activation of the innate immune system, as evidenced by the increased levels of pro‐inflammatory monocytes, macrophages, neutrophils, and cytokines such as IL‐1β, IL‐6, TNF‐α, and MCP‐1 [18, 19]. Given the central role of these inflammatory mediators in KD, we measured their levels in both the LCWE‐induced KD mouse model and the corresponding cell model. Our results demonstrated a significant increase in these cytokines in mouse serum and macrophage‐conditioned media following LCWE treatment. In addition, histological analysis revealed substantial infiltration of inflammatory cells around the aortic root and coronary arteries 14 days post‐LCWE injection. Collectively, these findings confirm that our animal and cell models effectively replicate the inflammatory features observed in human KD.

Furthermore, we found that LCWE stimulation of RAW264.7 macrophages increases Areg expression and secretion, which can be inhibited by the ADAM‐17 inhibitor TAPI‐1. ADAM‐17, a membrane‐binding enzyme, plays an important role in controlling inflammation and tissue regeneration. In the course of our experimental inquiry, the utilization of TAPI‐1, a chemical compound renowned for its capacity to impede ADAM‐17 activity, to investigate its effects on the cellular processes under examination. Meanwhile, TAPI‐1 exhibits a potent inhibitory activity against the expression of inflammatory mediators, including IL‐1, IL‐6, TNF‐α, and MCP‐1. However, knocking down ADAM‐17 by lentivirus, only IL‐6, TNF‐α, and Areg Inhibited significantly. TAPI‐1 is not a specific ADAM17 inhibitor, so that it was not applied at the level of KD animal models. In this part of the study, we also explored the effect of Areg on inflammatory cytokines. The results shown neither the increase nor depletion of Areg had any significant effect on the expression of IL‐1, IL‐6, TNF‐α, and MCP‐1.

Our study further explores the effects of Areg on EC function. Endothelial migration and proliferation are important to the formation and damage repair of vascular [20]. CCK8 and scratch assay were used to tested the proliferation and migration ability of MCAECs. In the vitro study, MCAECs treated with LCWE‐induced RAW‐CM showed a rise in the proliferative capability and a decreased migration capacity. Macrophages have both promoting and inhibiting effects on ECs, which may be due to the simultaneous expression of Areg and inflammatory cytokines. Areg plays a key role in restoring tissue integrity after infection or injury, partly because it is expressed rapidly at the site of tissue injury by infiltrating leukocytes [21]. Through the EGFR signaling pathway, Areg promote the proliferation and migration of a variety of cells, including ECs [22, 23]. We found the AKT and ERK signaling pathways are activated significantly in MCAECs treated with LCWE‐induced RAW‐CM. We hypothesize that Areg, derived from LCWE‐induced macrophage, is critical in the promotion of ECs proliferation as well as the activation of AKT and ERK signaling pathways.

Considering ADAM‐17 is a common enzyme for multiple inflammatory factors and Areg, its inhibitors alone cannot clarify the role of Areg in LCWE‐induced endothelial injury. Our work continues as follows. In this study, we found that the addition of Areg alone or in an additional EC injury model enhanced the proliferation and migration ability of MCAECs and activated the AKT and ERK signaling pathways. These results indicate that exogenous Areg can promote the function of both damaged and healthy coronary ECs. By blocking Areg secreted by macrophages with Areg‐specific antibodies, we found that MCAECs proliferation and migration were significantly inhibited in EC injury models, along with the AKT and ERK phosphorylation levels were reduced. These results confirmed that macrophage‐derived Areg had a protective function on injured ECs, as well as LCWE‐activated macrophages can promote the proliferation and migration of MCAECs by secreting Areg.

Our results align with previous studies demonstrating the tissue‐repairing and anti‐inflammatory properties of Areg. For example, Areg has been shown to promote wound healing and tissue regeneration in various models of injury. Additionally, Areg's role in modulating immune responses has been highlighted in studies showing its ability to suppress excessive inflammation. However, our study uniquely focuses on the context of KD and provides insights into the specific mechanisms through which Areg may contribute to EC repair in this disease setting.

## 5 Conclusions

In conclusion, our study provides novel insights into the expression and role of Areg in KD. We demonstrate that Areg levels are elevated in KD models and that Areg promotes the proliferation and migration of injured ECs through the AKT and ERK signaling pathways. These findings suggest that Areg may be a promising target for the diagnosis and treatment of KD, warranting further investigation.

## Author Contributions

**Jiawen Xu:** writing – original draft. **Yihua Jin:** writing – review and editing. **Min Wang:** data curation. **Yijing Tao:** investigation. **Yujia Wang:** writing – review and editing. **Fangqi Gong:** writing – review and editing.

## Conflicts of Interest

The authors declare no conflicts of interest.

## Data Availability

The data that support the findings of this study are available from the corresponding author upon reasonable request.
